# Genome-Wide Characterization, Expression, and Functional Analysis of Acyl-CoA-Binding Protein (ACBP) Gene Family in *Puccinellia tenuiflora*

**DOI:** 10.3390/plants14233551

**Published:** 2025-11-21

**Authors:** Hongxia Zheng, Ying Li, Sizhu Wang, Xin Ni, Xiaofeng Xu, Shaojun Dai

**Affiliations:** 1 Key Laboratory of Saline-Alkali Vegetation Ecology Restoration, Ministry of Education, College of Life Sciences, Northeast Forestry University, Harbin 150040, China; zhenghongxia2020@foxmail.com (H.Z.); ly7966@nefu.edu.cn (Y.L.); wangsizhu202510@163.com (S.W.); 13136869041@139.com (X.N.); 2 Development Center of Plant Germplasm Resources, College of Life Sciences, Shanghai Normal University, Shanghai 200234, China

**Keywords:** acyl-CoA-binding proteins (ACBPs), gene family analysis, salt response, *Puccinellia tenuiflora*

## Abstract

Acyl-CoA-binding proteins (ACBPs) possess a conserved acyl-CoA-binding (ACB) domain that facilitates binding to acyl-CoA esters. In addition to their typical role in lipid metabolism, plant ACBPs have been shown to participate in various physiological processes, such as membrane biogenesis, stress response pathways and plant immunity mechanisms. Here, we identified five *PutACBP* members in alkaligrass (*Puccinellia tenuiflora*), which were divided into four distinct classes based on a phylogenetic tree constructed from 86 *ACBP* genes from 12 plant species. Promoter analysis identified numerous *cis*-acting elements linked to abiotic stresses (e.g., light, drought, heat, and cold) and hormone responses. Expression profile analyses revealed that *PutACBPs* exhibit broad expression patterns across many organs and respond to salinity-alkali, cold, H_2_O_2_, and CdCl_2_ stresses. Transient expression of five PutACBP-GFPs in tobacco (*Nicotiana tabacum*) revealed PutACBP1 and PutACBP2 localized to the plasma membrane, cytoplasm, and cell nucleus, while PutACBP3, PutACBP4, and PutACBP5 localized around the plasma membrane and cytoplasm. Furthermore, heterologous constitutive expression of *PutACBP3* in *Arabidopsis* (*Arabidopsis thaliana*) enhanced the resistance of transgenic plants to salinity stress, possibly through alterations in the levels of lipid metabolism-related and stress-responsive genes. The *ACBP* gene family is highly conserved across different plant species. This study provides the first comprehensive genomic and functional characterization of the PutACBP family in alkaligrass, elucidating its evolutionary conservation, phylogenetic classification, and stress-response roles. Notably, overexpression of *PutACBP3* in *Arabidopsis* significantly enhanced salt tolerance, suggesting its critical function in salt-stress adaptation in alkaligrass.

## 1. Introduction

Lipids are essential for maintaining membrane stability, energy supply, and stress adaptation in plants [1]. Acyl-CoA-binding proteins (ACBPs), characterized by conserved acyl-CoA-binding (ACB) domains, function as lipid carrier proteins that mediate lipid binding, transport, and metabolism across cellular compartments [2]. ACBPs were initially identified in mammals as Diazepam Binding Inhibitor (DBI) [3,4], while plant ACBPs were first reported in oilseed rape (*Brassica napus*) [5]. Subsequently, ACBP families have been identified at least in 27 plant species, ranging phylogenetically from algae, moss, ferns, and gymnosperms to angiosperms [6,7]. Based on their size and domain architecture, plant ACBPs are classified into four classes: Class I (single ACB domain), Class II (ACB domain with C-terminal ankyrin repeats), Class III (extended proteins with a C-terminal ACB domain), and Class IV (multi-domain proteins with C-terminal kelch motifs) [8,9,10,11].

ACBPs are evolutionarily conserved across eukaryotes and play diverse roles in lipid metabolism, membrane biosynthesis, and stress adaptation [12,13,14]. For instance, yeast ACBPs are critical for plasma membrane assembly, and their mutations disrupt key pathways such as fatty acid biosynthesis, glycerol metabolism, and stress responses [15,16]. In plants, *ACBPs* exhibit evolutionarily conserved isoform specialization in lipid binding [17], as evidenced by systematic characterization of *Arabidopsis* (*Arabidopsis thaliana*) isoforms (AtACBP1–6). AtACBP1 and AtACBP2 proteins preferentially recognize C18:2-CoA, C18:3-CoA esters, phosphatidylcholine (PC), and phosphatidic acid (PA) [18,19,20,21], while AtACBP3 exhibits broader specificity for arachidonyl-CoA (C20:4), PC, and phosphatidylethanolamine (PE) [21,22]. AtACBP4 and AtACBP5 show a distinct preference for C18:1-CoA and PC [23,24,25,26], whereas AtACBP6 selectively binds 16:0-CoA and 18:2-CoA [27]. These isoform-specific binding profiles establish plant ACBPs as modular transporters that orchestrate compartmentalized lipid trafficking via precision-tuned molecular recognition.

Soil salinity impairs plant growth by inducing osmotic, ionic, and oxidative stress [28,29,30,31,32,33]. Salt stress signaling involves phospholipids such as PA and the salt overly sensitive (SOS) pathway, in which PA activates mitogen-activated protein kinase 6 (MPK6) to phosphorylate SOS1, thereby regulating Na^+^ homeostasis [34,35,36,37,38,39]. *ACBPs* play critical roles in salt stress adaptation. For example, *Chlorella* sp. *ChACBP1* enhances salinity tolerance, and its overexpression in yeast and *Arabidopsis* likely improves stress resilience via phospholipid metabolism [40]. In *Arabidopsis*, *AtACBP1* overexpression increased sensitivity to NaCl during seed germination and seedling establishment, whereas the *atacbp1* mutant showed reduced sensitivity during germination [41]. Conversely, overexpression of *OsACBP4* in transgenic rice (*Oryza sativa*) and *AtACBP2* in transgenic *Arabidopsis* conferred enhanced salt tolerance in both species [42]. In maize (*Zea mays*), NaCl induces expression of *ZmACBP1* and *ZmACBP3*, and overexpression of *ZmACBP3* (*ZmACBP3-OEs*) in *Arabidopsis* promoted better growth and longer roots under salt stress. *ZmACBP3-OEs* also exhibited significantly upregulated expression of lipid metabolism genes (*FAD2*, *DGAT*, *PLA2*, *PLC3*, and *ACX*) and stress-responsive genes (*COR47*, *AREB1*, *RAB*, *ABI1*, *RD29A*, and *RD29B*) compared to WT (wild-type) plants, suggesting *ZmACBP3* enhances tolerance by modulating lipid metabolism and activating stress-responsive gene expression [7]. Similarly, soybean (*Glycine max*) *GmACBPs* exhibit differential expression under salt stress, suggesting functional diversification among *ACBP* family members [43].

Beyond salinity, *ACBPs* also contribute to diverse stress responses. *AtACBP1* and *AtACBP2* in *Arabidopsis* facilitate membrane repair by binding to phospholipid precursors under heavy metal stress [20,44]. Additionally, *AtACBP2*-overexpressing plants exhibit increased sensitivity to abscisic acid (ABA) during seed germination and development, where ABA-mediated reactive oxygen species (ROS) accumulation in guard cells enhances drought tolerance by promoting stomatal closure [45]. Furthermore, overexpression of *AtACBP3* in *Arabidopsis* enhances disease resistance by upregulating pathogen-associated genes, increasing cell mortality, and accumulating hydrogen peroxide [46]. These findings underscore the importance of *ACBPs* in plant stress responses, particularly through their roles in lipid signaling, membrane repair, and ABA-mediated stress adaptation.

Alkaligrass (*Puccinellia tenuiflora*) is a halophytic monocot forage grass that thrives in saline-alkali soils, making it an ideal model for studying salt adaptation mechanisms [47]. Comparative genomic and transcriptomic analyses have identified thousands of salinity-responsive genes in alkaligrass [48]. Quantitative proteomic studies have systematically mapped stress-induced changes in protein abundance and post-translational modifications under diverse stressors, including salts (NaCl, Na_2_CO_3_, and NaHCO_3_), low temperatures, and oxidative stress [49,50,51,52,53,54,55,56]. Integrated multi-omics data reveal that alkaligrass employs unique adaptive strategies for salinity tolerance, including the proteins involved in fatty acid metabolism particularly through dynamic regulation of its robust antioxidant systems for ROS scavenging [49]. Notably, four root *PutACBPs* (*PutACBP1*-*PutACBP4*) and two leaf *PutACBPs* (*PutACBP1* and *PutACBP3*) are differentially expressed under NaCl, Na_2_CO_3_, and NaHCO_3_ stresses [48], suggesting their involvement in lipid-mediated stress responses. However, a comprehensive analysis of the *ACBP* gene family in alkaligrass, including its phylogenetic evolution, expression dynamics and functional validation, has not yet been conducted.

In this study, we identified five *PutACBP* genes through whole-genome screening. We systematically analyzed the phylogenetic evolution, organ-specific and stress-responsive expression profiles, and association with agronomic traits of *PutACBPs*. Our findings demonstrate that *PutACBP3* enhances salinity resistance through phospholipid-mediated membrane remodeling and ROS homeostasis, providing novel insights into halophyte stress adaptation mechanisms.

## 2. Results

### 2.1. Identification and Phylogenetic Analysis of ACBP Genes in Alkaligrass

To identify ACBPs in alkaligrass, we performed a BLASTp search using known ACBP sequences from *Arabidopsis* and rice. This initial search identified 15 candidate genes predicted to encode ACBPs. To validate these candidates and confirm the presence of the defining acyl-CoA-binding domain, we screened all 15 sequences against the conserved ACB domain (PF00887). Furthermore, candidates were analyzed for the presence of Ankyrin and Kelch domains characteristic of specific ACBP classes. This combined domain analysis confirmed five bona fide *ACBP* genes in alkaligrass, designated PutACBP1 to PutACBP5 with locus identifiers Pt_Chr0101998, Pt_Chr0106094, Pt_Chr0300051, Pt_Chr0402341, and Pt_Chr0402344 (Table S1). The deducted PutACBPs range in size from 89 to 655 amino acids (AA) and exhibit isoelectric points (pI) between 4.45 and 5.08. All five proteins are hydrophilic, as indicated by negative Grand Average of Hydropathy (GRAVY) values. PutACBP1 and PutACBP2 show high sequence similarity. Detailed characteristics, including molecular weight (MW), coding sequence (CDS) length, AA number, pI, GRAVY, subfamily classification, and the predicted subcellular localization for each gene, are provided in Table S1.

**Figure 1:**
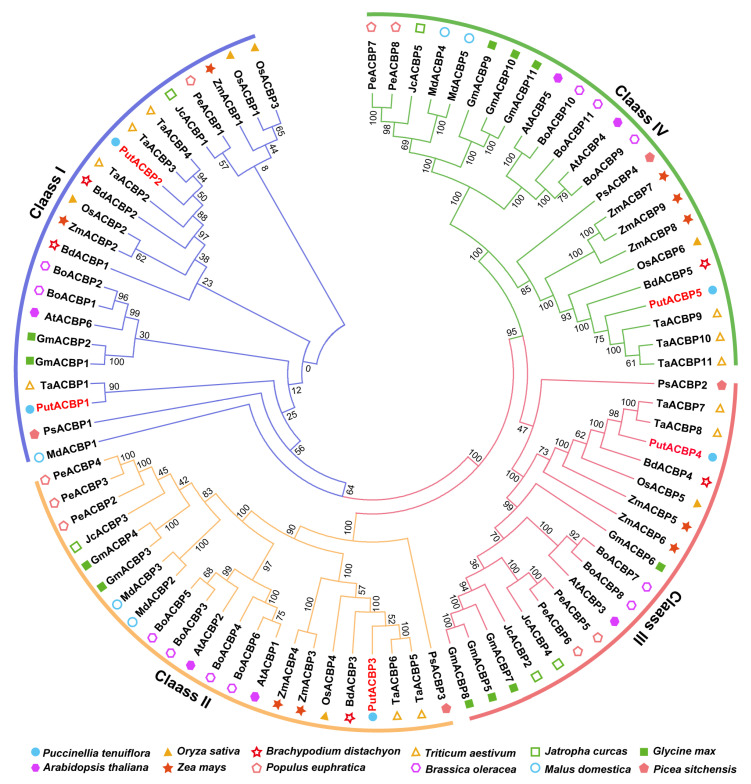
Phylogenetic tree of acyl-CoA-binding proteins (ACBPs) from alkaligrass (*Puccinellia tenuiflora*) and other representative plant species. Alignments of 86 ACBPs in alkaligrass, rice (*Oryza sativa*), *Brachypodium distachyon*, wheat (*Triticum aestivum*), leper tree (*Jatropha curcas*), soybean (*Glycine max*), *Arabidopsis*, maize (*Zea mays*), poplar (*Populus euphratica*), cabbage (*Brassica oleracea*), apple (*Malus domestica*), and spruce (*Picea sitchensis*) were performed using the default parameter of ClustalW and the phylogenetic tree was constructed using the Neighbor-Joining tree method with 1000 bootstrap replicates in MEGA 7.0 software. These ACBPs were divided into four classes. Different colored circles represent different plant species.

To classify the identified PutACBPs and determine their evolutionary relationships, we constructed a phylogenetic tree using the neighbor-joining method. The tree was based on the alignment of full-length ACBP sequences from alkaligrass and *Arabidopsis* (Figure 1). Phylogenetic analysis revealed that the PutACBPs share high similarity with rice OsACBPs and cluster into the four established plant ACBP classes. Specifically, PutACBP1 and PutACBP2 group within Class I (characterized by a single ACB domain). PutACBP3, PutACBP4, and PutACBP5 were classified into Class II (ACB domain + ankyrin repeats), Class III (large protein with C-terminal ACB domain), and Class IV (multi-domain with kelch motifs), respectively. This provides a crucial foundation for subsequent functional characterization of these ACBPs in abiotic stress responses.

### 2.2. Evolutionary Genomics Analysis Reveals Chromosomal Distribution and Conservation of ACBP Genes in Alkaligrass

**Figure 2:**
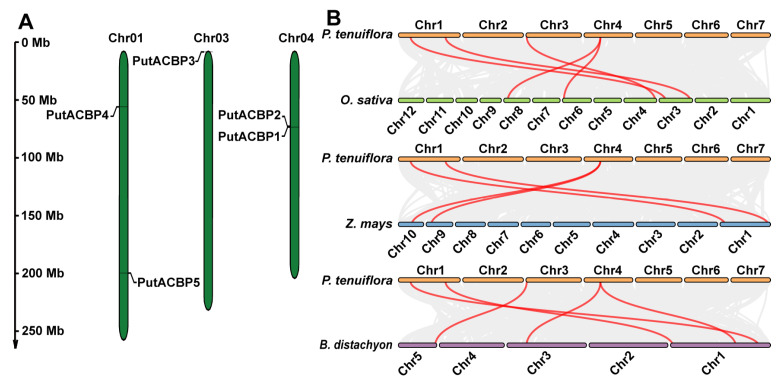
Distribution of *PutACBPs* on chromosomes and collinearity analysis of *ACBPs* between alkaligrass and other plant species. (**A**) Chromosomal localization of the *PutACBP* genes. The diagram was drawn using the MapGene2Chromorome V2 (v 2.1), and five *PutACBP* genes were located on three chromosomes. Chromosomal distances are given in Mb. (**B**) Collinearity analysis of *ACBPs* among alkaligrass, rice, maize, and *B. distachyon*. Gray lines in the background indicated the collinear blocks within alkaligrass and other plant genomes, while the red lines highlighted the syntenic *ACBP* gene pairs.

Analysis of chromosomal distribution revealed that the five *PutACBP* genes are located on three chromosomes (1, 3, and 4) of alkaligrass, displaying an uneven distribution pattern (Figure 2A). *PutACBP1* and *PutACBP2* are localized in close proximity on the same chromosome, suggesting that they may have arisen from a common ancestral gene and potentially share similar structures and functions. This non-random localization mirrors observations in *Arabidopsis* and rice, where *ACBP* genes are also unevenly distributed across their respective genomes [9,57]. These findings suggest that the chromosomal arrangement of *ACBP* genes may be a conserved feature across plant species.

To elucidate evolutionary relationships within the ACBP family, we performed collinearity analysis using homologous *ACBP* genes from alkaligrass, rice, maize, and *B. distachyon*. The analysis identified five, four, and five homologous gene pairs between alkaligrass *ACBPs* and their counterparts in rice, maize, and *B. distachyon*, respectively (Figure 2B). Notably, no maize ortholog was identified for *PutACBP3*. This absence suggests lineage-specific evolutionary divergence, potentially linked to extensive gene fractionation following the ancestral tetraploidization event in maize, which may have led to the loss of this specific ancestral *ACBP* homolog. In contrast, *B. distachyon* and rice appear to have retained orthologs of *PutACBP3* under stronger purifying selection, implying functional conservation potentially critical for niche adaptation. The conservation of *PutACBP3* orthologs in *B. distachyon* and rice further suggests its involvement in fundamental cellular processes. Its loss in maize may have been evolutionarily permissible due to functional redundancy, potentially compensated by paralogous genes or alternative metabolic pathways. Collectively, the collinearity patterns indicate that *PutACBP* genes exhibit relatively greater evolutionary conservation with *B. distachyon* and rice than with maize.

### 2.3. Comparison in Architecture of ACBP Domain from Arabidopsis, Rice, and Alkaligrass

**Figure 3:**
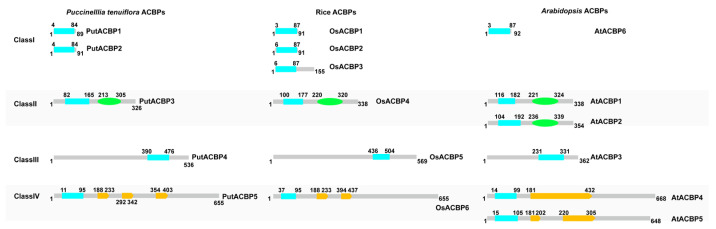
Architecture of ACBPs in alkaligrass. Comparation of the architecture of ACBPs among alkaligrass, rice, and *Arabidopsis*. Blue indicates the acyl-CoA-binding (ACB) domain, green indicates the ankyrin repeats (Ankyrin) domain, and yellow indicates the Kelch domain.

According to different domains and amino acid lengths, the five ACBPs were divided into four subfamilies (Figure 3). All ACBPs contain an ACB domain, and some of them also contain the Ankyrin domain or the Kelch domain. PutACBP1 and PutACBP2 belong to Small-ACBP in Class I and contain only one ACB domain. PutACBP3 contains an ACB domain and an Ankyrin domain and was classified as ankyrin-repeat ACBPs (ANK-ACBP) in Class II. PutACBP4 was categorized as Large-ACBP in Class III and only contains one ACB domain in the C-terminal. PutACBP5 belongs to Kelch-ACBPs in Class IV and contains one ACB domain in the N-terminal and three Kelch domains in the center (Figure 3).

When compared PutACBPs with those from *Arabidopsis* and rice, we found that the AA sequences of PutACBPs are more similar with those from rice. In *Arabidopsis*, Class II and Class IV contain two ACBPs, while rice and alkaligrass contain only one ACBP gene (Figure 3, Table S4). Collectively, PutACBPs show higher similarity to rice homologs.

### 2.4. Analysis of Gene Structure, Cis-Acting Elements, and Conserved Motif Distribution

**Figure 4:**
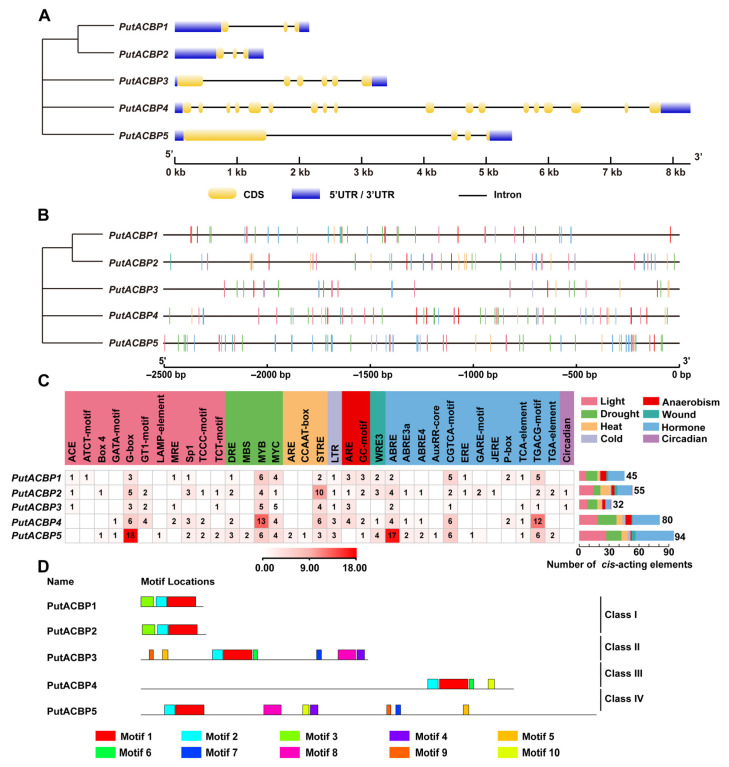
Structure, *cis*-acting elements, and conversed protein motifs of PutACBPs. (**A**) Phylogenetic tree and gene structure analyses of *PutACBPs*. The UTR, exon and intron were represented by blue rectangle, yellow rectangle, and black line, respectively. (**B**) Predicted *cis*-acting elements in the promoter regions of *PutACBPs*. (**C**) The names and numbers of *cis*-acting elements in *PutACBP* promoters. The heatmap in grid and the color columns indicated the numbers of *cis*-acting elements. ABRE: ABA responsive element; ARE: Anaerobic-responsive element; DRE: Dehydration-responsive element; JERE: Jasmonate and/or elicitor responsive element; LTR: Low temperature-responsive element; MBS: MYB-binding site; MYB: V-myb avian myeloblastosis viral oncogene homolog; MYC: Myelocytomatosis; WRE3: Wound response element 3. (**D**) Conversed motif analysis in PutACBPs. The conserved motifs were predicted using MEME motif search analysis, and the maximum number parameter was set to ten. Various motifs were represented by different colors. The sequence information for each motif was provided in Table S2.

Gene structure analysis of the five *PutACBPs* was performed using the Gene Structure Display Server (http://gsds.cbi.pku.edu.cn, accessed on 27 June 2025). Based on genomic sequences, exon numbers were largely conserved within each *PutACBP* subfamily, while introns varied in both length and position (Figure 4A).

To investigate the potential functions of *PutACBPs*, a prediction of *cis*-acting elements was made in five *PutACBP* promoter regions and a schematic map was created of the prevalent distributions of stress and hormone responsive *cis*-acting elements (Figure 4B). A total of 306 *cis*-acting elements in *PutACBPs* were predicted, which were associated with various abiotic stresses, including light (74 elements), drought (61), heat (28), anaerobic conditions (19), wounding (10), cold (9), circadian (2), and hormones (102) (Figure 4C). Among these, certain elements responsive to drought (e.g., DRE, MBS, MYB, and MYC), light-responsive elements (e.g., ACE, ATCT-motif, Box4, GATA-motif, G-box, GT1-motif, LAMP-element, MRE, Sp1, TCCC-motif, and TCT-motif), heat-responsive elements (e.g., ARE, CCAAT-box, and STRE), and phytohormones together account for a significant portion of the total in each of the *PutACBPs*, some of which are conserved among the five *PutACBPs* (Figure 4C). These findings suggest that *PutACBPs* play vital roles in responding to drought, light, heat, and hormonal signals. It is noteworthy that a total of 102 hormone-responsive elements were detected, including 29 ABA (also known as ABRE), 40 methyl jasmonate (MeJA) (specifically TGACG and CGTCA motifs), and 4 salicylic acid (SA) (identified as TCA element), 11 gibberellin (GA) (specifically P-box and TATC-box), and 5 auxin (specifically TGA-element and AuxRR-core) (Figure 4C). The conserved elements found in the promoter region indicate that *PutACBPs* play a crucial role in responding to abiotic stresses and phytohormone treatments.

Motif analysis using the MEME Suite systematically identified ten conserved structural elements (motifs 1–10) across PutACBP isoforms (Figure 4D and Figure S1). Regarding motif distribution per class (Figure 4D): Class I contained three motifs, while Class II, III, and IV contained eight, four, and eight motifs, respectively. Importantly, Motif 1 contains the signature ACB domain essential for protein function. Subfamily members exhibited similar motif composition and arrangement (Figure 4D). For instance, motif 1 and motif 2 were present in all *PutACBP* genes, while motif 3 was specific to Class I. Motifs 4, 5, 7, 8, and 9 were found in Class II and Class IV. Motif 6 was observed in Class II and Class III, and motif 10 was present in Class III and Class IV. The distinct distribution of these motifs across subfamilies suggest functional diversity.

### 2.5. Expression Pattern of PutACBP Genes in Different Organs

**Figure 5:**
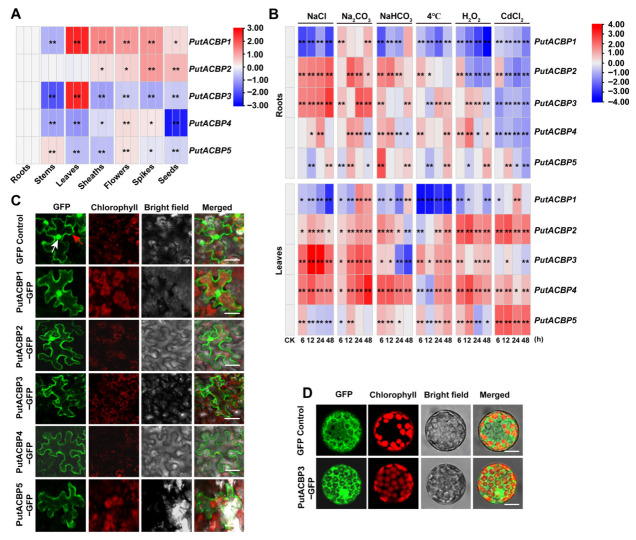
Organ specific expression, different stress expression, and subcellular localization of PutACBPs. (**A**) Organ specific expression *PutACBPs* were analyzed in roots, stems, leaves, sheaths, flowers, spikes, and seeds using RT-qPCR analysis. (**B**) Different stress expression patterns in roots and leaves of *PutACBPs*. The heatmap of *PutACBPs* expression based on the RT-qPCR data standardized by log2 conversion, blue indicates down regulated expression and red indicates upregulated expression. (**C**) Subcellular localization of PutACBPs in tobacco epidermal cells. The epidermal cells of tobacco leaves were examined under a confocal laser scanning microscope. Chloroplasts were visualized by chlorophyll autofluorescence. The red arrow indicates the cell membrane, while the white arrow indicates the cell nucleus. Scale bars = 25 μm. (**D**) Subcellular localization of PutACBP3 in *Arabidopsis* protoplasm cells. The protoplasts were extracted from *Arabidopsis* transgenic seedlings and examined under a confocal laser scanning microscope. Chloroplasts were visualized by chlorophyll autofluorescence. Scale bars = 10 μm. Significant differences compared to the control group were determined by Student’s *t*-test, ** *p* < 0.01, and * *p* < 0.05.

To investigate the organ-specific expression profiles of *PutACBP* genes, we performed RT-qPCR analysis across seven plant organs. The expression of *PutACBP* genes in different organs (i.e., roots, stems, leaves, sheaths, flowers, spikes, and seeds) were analyzed by RT-qPCR. The results showed that five *PutACBP* genes were detected in all the organs (Figure 5A, Table S6). *PutACBP1* and *PutACBP3* exhibited high expression levels in leaves. *PutACBP1* expression was lower in roots and stems compared to other organs, while *PutACBP2* showed reduced expression in roots, stems, and leaves (Figure 5A). *PutACBP4* expression was lowest in seeds relative to other organs (Figure 5A). These distinct expression patterns suggest functional diversification within the *PutACBP* family.

### 2.6. Expression Profiling of PutACBP Genes in Response to Abiotic Stress

To investigate the potential role of *PutACBP* genes under abiotic stress, their expression was assessed via RT-qPCR following treatment with salts (200 mM NaCl, 75 mM Na_2_CO_3_, 100 mM NaHCO_3_), low temperature (4 °C), oxidation (10 mM H_2_O_2_), and heavy metal (160 μM CdCl_2_) stresses (Figure 5B, Table S7). Under NaCl stress, *PutACBP1* was down-regulated in leaves and roots, while *PutACBP2*, *PutACBP3*, and *PutACBP4* in leaves, as well as PutACBP2 and PutACBP3 in roots, were significantly up-regulated (Figure 5B). Similarly, Na_2_CO_3_ and NaHCO_3_ stresses induced *PutACBP2*, *PutACBP3*, and *PutACBP4* expression in leaves or roots at certain timepoints (Figure 5B). Furthermore, low temperature (4 °C) induced *PutACBP2* to *PutACBP5* expression at certain timepoints, but significantly reduced *PutACBP1* in roots and leaves. Oxidative stress (H_2_O_2_) reduced *PutACBP1* in roots and leaves while inducing *PutACBP2* to *PutACBP5* in leaves. In addition, CdCl_2_ induced *PutACBP2*, *PutACBP4*, and *PutACBP5* in leaves but reduced the expression of all *PutACBPs* in roots (Figure 5B). These results indicate that *PutACBPs* play important roles in alkaligrass tolerance to different abiotic stresses.

### 2.7. Subcellular Localization of ACBPs in Alkaligrass

To determine the subcellular localization of PutACBPs in alkaligrass, we performed transient transformation in tobacco leaves using *35S::GFP* and *35S::PutACBP-GFP* constructs, and stable transformation of *35S::PutACBP3-GFP* in *Arabidopsis* seedlings. In tobacco epidermal cells, PutACBP1-GFP and PutACBP2-GFP displayed fluorescence in the plasma membrane, cytoplasm, and nucleus. In contrast, fluorescence signals from the PutACBP3-GFP, PutACBP4-GFP, and PutACBP5-GFP proteins were primarily localized to the plasma membrane and cytoplasm (Figure 5C). This membrane-associated localization pattern of PutACBP3-GFP was further supported by experiments in *Arabidopsis* protoplasts (Figure 5D).

### 2.8. PutACBP3 Is Essential for Seedling Salt Tolerance

**Figure 6:**
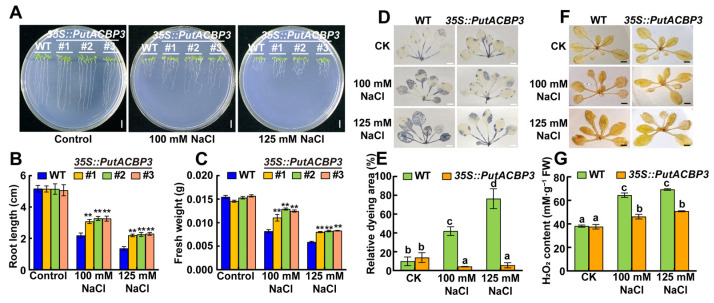
The phenotypes of WT (wild-type) and *PutACBP3*-overexpressing *Arabidopsis* plants. (**A**) Seedlings phenotypes of WT, *35S::PutACBP3* (#1, #2, and #3) under normal condition (1/2 MS medium), 100 mM NaCl, and 125 mM NaCl stresses for ten days. The scale bar is 2 cm. (**B**) Root growth WT and three *35S::PutACBP3* alleles. (**C**) Fresh weight of WT and three *35S::PutACBP3* alleles under normal conditions (1/2 MS medium) NaCl stress conditions. Each value represents the mean ± SD (*n* = 15). Error bars indicate SD. Student’s *t*-test. ** *p* < 0.01. (**D**) Visualization of O^2·−^ by NBT staining, in leaves of three-week-old *Arabidopsis* WT and transgenic seedlings under normal condition (1/2 MS), 100 mM NaCl, and 125 mM NaCl stresses for 48 h. The blue colors show the accumulation of O^2·−^. The scale bar = 0.5 cm. (**E**) The statistics of dyeing area of leaves in (**D**). The values were presented as mean ± SE (*n* = 3). (**F**) Visualization of H_2_O_2_ by DAB staining, in leaves of three-week-old *Arabidopsis* WT and transgenic seedlings under normal condition (1/2 MS), 100 mM NaCl, and 125 mM NaCl stresses for 48 h. The brown colors show the accumulation of H_2_O_2_. The scale bar = 0.5 cm. (**G**) H_2_O_2_ contents in 3-week-old *Arabidopsis* WT and transgenic seedlings under normal condition (1/2 MS), 100 mM NaCl, and 125 mM NaCl stresses for 48 h. The values were presented as mean ± SD (*n* = 3). One-way ANOVA was used for statistical analyses in (**E**,**G**). Different letters indicate significant differences.

To investigate the function of *PutACBP3* in response to salt stress, we constructed *Arabidopsis PutACBP3*-overexprssing seedlings and compared their phenotypes with WT. Under salt stress (100 mM NaCl and 125 mM NaCl), *PutACBP3*-overexprssing seedlings exhibited significantly longer roots and higher fresh weight than those of WT (Figure 6A–C), which indicates *PutACBP3* enhanced the salt tolerance.

The NBT and DAB staining was applied to detect O^2−^ accumulation and H_2_O_2_ content in the leaves, respectively. Both O^2−^ accumulation and H_2_O_2_ contents were much lower in transgenic seedlings than the WT under the treatments of NaCl (Figure 6D–F). The H_2_O_2_ contents in transgenic seedlings were not different from that in WT seedlings in normal condition. However, the H_2_O_2_ contents were obviously lower in all the transgenic lines under NaCl treatment (Figure 6G).

### 2.9. Phosphatidylcholine-Specific Binding of PutACBP3 Associates with PLDδ-Mediated Membrane Remodeling for Enhanced Salinity Tolerance

**Figure 7:**
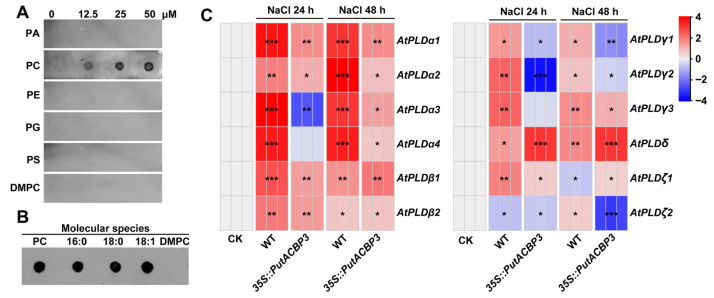
Recombinant protein His-PutACBP3 binding to phospholipids in vitro and expression profiles of *PLD* genes in *PutACBP3*-overexpressing *Arabidopsis* seedlings. (**A**) His-PutACBP3 binds to phosphatidylcholine (PC) on filters. Various concentrations (0, 12.5, 25, and 50 μM) of lipids including phosphatidic acid (PA), PC, phosphatidylethanolamine (PE), phosphatidyl glycerol (PG), phosphatidylserine (PS), and Dimyristoylphosphatidylcholine (DMPC) were spotted onto nitrocellulose and incubated with 1 μg of purified His-PutACBP3 proteins. The His-PutACBP3 binding was detected by immunoblotting with HRP-conjugated anti-penta-His antibodies. (**B**) Effect of PC acyl species on His-PutACBP3/PC binding. 50 μM lipids (PC, 16:0-PC, 18:0-PC, 18:1-PC or DMPC) were spotted onto nitrocellulose and then were incubated with 1 μg ml^−1^ of purified His-PutACBP3 protein. The His-PutACBP3 lipid binding was detected by immunoblotting with HRP-conjugated anti-penta-His antibodies. (**C**) Expression profiles of the 12 *AtPLD* genes in WT and *PutACBP3*-overexpressing *Arabidopsis* seedlings illustrated by a heat map under normal conditions (1/2 MS medium), and NaCl stress conditions. Significant differences compared to the control group were determined by Student’s *t*-test, *** *p* < 0.001, ** *p* < 0.01, and * *p* < 0.05.

To elucidate the biochemical properties and physiological function of PutACBP3, we conducted lipid-binding assays and stress response analyses. Recombinant His-PutACBP3 protein migrated at the predicted molecular mass (~54 kDa) on Coomassie-stained SDS-PAGE gels (Figure S2). In vitro lipid binding assays revealed a specific affinity of PutACBP3 for PC, including the variants 16:0-PC, 18:0-PC, and 18:1-PC. In contrast, no significant binding was detected to phosphatidylserine (PS), PA, PE, phosphatidylglycerol (PG), or dimyristoylphosphatidylcholine (DMPC) (Figure 7A,B).

Given this specific PC-binding activity and the enhanced salinity tolerance observed in *PutACBP3*-overexpressing *Arabidopsis* (Figure 6), we investigated its potential link to phospholipid signaling pathways known to be involved in stress responses. Considering the established roles of phospholipase D (PLD) isoforms in stress signaling and membrane lipid remodeling, we analyzed the expression of *Arabidopsis PLD* genes in WT and *35S::PutACBP3* seedlings under 100 mM NaCl treatment. RT-qPCR revealed that the expression level of *AtPLDδ* was significantly higher in *35S::PutACBP3* seedlings compared to WT at both 24 h and 48 h after salt treatments (Figure 7C, Table S8). These results suggest that the enhanced salt tolerance conferred by *PutACBP3* overexpression may be associated with elevated *PLDδ* expression. This observation is consistent with a model where PutACBP3, through its specific interaction with PC, influences phospholipid metabolism and signaling, potentially facilitating PLDδ-mediated membrane remodeling processes critical for salinity adaptation.

## 3. Discussion

### 3.1. Evolutionary Conservation and Functional Diversification of ACBPs

ACBPs have been functionally annotated in several higher plants, including *Arabidopsis*, rice, and *B. napus* [57]. Phylogenetic analyses indicate that ACBPs are conserved across all plants, including green microalgae (e.g., *Chlorella* sp.), bryophytes (e.g., *Physcomitrella*), and vascular plants [7], suggesting an origin in early photosynthetic eukaryotes. Our phylogenetic analysis revealed that the five PutACBPs were grouped into four subfamilies (Figure 1), consistent with finding in *Arabidopsis* and rice [9]. Comparative analyses further reveal species-specific variations in the types and numbers of ACBP genes (Figure 1), potentially reflecting functional specialization across plant lineages. This systematic characterization sheds light on the evolutionary diversification and stress adaptation mechanisms of ACBPs in plants adapted to extreme environments. Sequence alignment analysis showed that PutACBP shares similar functional domains with ACBP homologs from rice (Figure 3). Gene structure analysis of ACBP genes indicated that genes within the same branch generally possessed similar exon-intron structures (Figure 4A). In addition, the motif analysis of ACBPs also found that the ACBPs that were clustered in the same subfamily shared a similar motif composition (Figure 4D). The high sequence similarity observed across species suggests that these genes are likely to share conserved functions.

### 3.2. Stress-Responsive Regulation of PutACBPs

*Cis*-acting elements are essential in many biological processes and stress responses [58]. The G-Box, ARE, P-Box, ABRE, and CGTCA-motif are *cis*-acting elements that respond to light, heat, and hormonal induction [3,59,60,61,62,63,64,65]. We found that G-Box, ARE, P-Box, ABRE, and CGTCA-motifs exit in the promoter of *PutACBP* genes (Figure 4B), implying that *PutACBPs* might respond to light, heat and hormone treatments. However, unlike Class II *PtACBP3*, which lacks the hypoxia-specific inducible GC-motif, members of Classes I, III, and IV appear to participate in plant responses to abiotic stress and hypoxia, a functional similarity shared with Class III *AtACBP3* [66]. In our study, both *PutACBP1* and *PutACBP2* were classified into Class I. Promoter analysis revealed that they contain similar *cis*-acting elements, suggesting functional overlap in certain biological processes. In contrast, previous studies have shown that overexpression of *OsACBP4* and *AtACBP2*, both Class II members, enhances salt tolerance in rice and *Arabidopsis*, respectively [42]. Consistent with these findings, our results demonstrate that overexpression of *PutACBP3*, another Class II protein, also improves salt tolerance in *Arabidopsis* (Figure 6). Notably, all three genes contain stress-related *cis*-elements in their promoters. Together, these observations support the hypothesis that Class II *ACBPs* play a conserved and important role in the plant salt stress response.

This study showed that the expressions of *PutACBP2* and *PutACBP3* were the highest in the leaves of alkaligrass (Figure 5A), while previous studies showed that the expression levels of *AtACBP1* and *AtACBP2* were higher in the leaves of *Arabidopsis* [26,44,67]. The expression level of *OsACBP4* in rice leaves was about eight times that in seeds [9]. We found that the expression of *PutACBP1* was the highest in the spike, *PutACBP4* was the highest in the flower, and *PutACBP5* was the highest in the stem (Figure 5A). In *Arabidopsis*, *AtACBP3* was highly expressed in flowers and vegetative tissues, suggesting that extracellular localization of *AtACBP3* plays a role in plant defense [68], and *OsACBP5* was highly expressed at reproductive stage [57].

*PutACBPs* genes were mostly upregulated in roots and leaves under different stresses (Figure 5B), suggesting that *PutACBPs* may be involved in responses to salt, alkaline, low temperature, oxidation, and heavy metal stresses. Previous studies have shown that *OsACBP5* plays an important role in salt stress, drought stress, pathogen stress and physical damage response [9]. We propose that *PutACBPs* could facilitate improving the repair ability of cell membrane and energy supply.

### 3.3. PutACBP3 Enhances Salinity Tolerance via PC Binding and PLDδ Activation

We found that SOD activity was enhanced but ROS accumulation was reduced in *PutACBP3*-overexpressing *Arabidopsis* lines (Figure 6D–G). Moreover, PutACBP3 has unique PC binding specificity (Figure 7A,B). This indicates its role in PC-mediated membrane stabilization and ROS scavenging. This mechanism integrates lipid trafficking with oxidative stress mitigation under salinity [36,56].

ACBP-mediated lipid modulation likely serves as a critical interface between stress perception and physiological adaptation. The observed variations in *ACBP* expression patterns under stress conditions could drive dynamic changes in lipid abundance and composition. For instance, *Arabidopsis AtACBP6* overexpression elevates *PLDδ* expression during freezing stress [27], mirroring our finding that *PutACBP3* overexpression upregulates *PLDδ* under salinity (Figure 7C). PLDδ catalyzes phospholipid hydrolysis to PA, a central lipid mediator in osmotic stress responses, ABA signaling, and pathogen defense [44]. In this study, *PutACBP3* transgenic *Arabidopsis* exhibited enhanced salt tolerance (Figure 6), likely through initiating lipid-dependent signaling Via PA production and/or modulating membrane architecture through PC binding. While PLDδ upregulation in *PutACBP3*-overexpressing lines suggests PC-derived PA signaling (Figure 7C), future lipidomic studies are needed to quantify PA flux and clarify its role in SOS pathway activation [37,39]. Notably, PutACBP3 and AtACBP1 exhibit distinct lipid-binding specificities: PutACBP3 preferentially binds to PC, whereas AtACBP1 has a higher affinity for PA [45], may determine pathway selectivity across species. This evolutionary divergence highlights how monocots like alkaligrass have uniquely optimized lipid-mediated signaling for extreme environments. This lipid-mediated signal amplification regulates stress-related gene expression, thereby positioning *PutACBP3* as a key node in halophyte salt adaptation.

## 4. Materials and Methods

### 4.1. Identification of ACBP Family Genes in Alkaligrass

To identify PutACBPs, the protein database of Alkaligrass was downloaded from http://www.xhhuanglab.cn/data/alkaligrass.html (accessed on 20 June 2025) [48], and Hidden Markov Model (HMM) profile file (PF00887) was downloaded from the Pfam database (http://pfam.xfam.org, accessed on 20 June 2025) [69]. The HMM file was exploited as a query to identify PutACBPs in the alkaligrass protein database using the hmmer search command of the HMMER (version 3.0) software. AtACBPs (AT5G53470, AT4G27780, AT4G24230, AT3G05420, AT5G27630, and AT1G31812) were used as the query sequence in a BlastP search against the alkaligrass genome database online. The sequences obtained by the above two methods were used for the subsequent selection, and the screening criteria were based on the ACB domain at the N-terminus, ankyrin (ANK) domain, and the Kelch domain at the C-terminus [9]. Peptide length, MW, and pI of each PutACBP were calculated using the online ExPASy program (https://www.expasy.org, accessed on 20 June 2025) [70]. Detailed information of PutACBPs can be found in Table S1. The CDS and protein sequences of PutACBPs were displayed in Tables S2 and S3.

### 4.2. Phylogenetic Analysis of PutACBP Genes

To investigate the phylogenetic relationships of the *ACBP* gene families among 12 plants. ACBPs were aligned using the BioEdit program. A neighbor-joining (NJ) phylogenetic tree was constructed for these proteins with MEGA 7.0 software (https://www.megasoftware.net, accessed on 22 June 2025) [71]. Bootstrapping was performed with 1000 repetitions. The protein sequences of ACBPs were displayed in Table S3.

### 4.3. Chromosomal Localization of the PutACBP Genes

The chromosomal localization of the *PutACBP* genes were obtained from http://www.xhhuanglab.cn/data/alkaligrass.html (accessed on 25 June 2025) [48], and the diagram was drawn using the MapInspect software (http://mg2c.iask.in/mg2c_v2.0, accessed on 25 June 2025).

### 4.4. Collinearity Analysis of ACBPs Between Alkaligrass and Other Plants

The collinearity analysis of *ACBPs* between the rice, maize, and *B. distachyon* was verified and visualized using One Stem MCScanX and Dual Systeny Plot for MCScanX in TBtools (v2.322) software, respectively [72].

### 4.5. Exon/Intron Structure, Conserved Motifs and Promoter Cis-Acting Element Analysis

The distribution patterns of exons and introns in *PutACBP* genes were predicted using the Gene Structure Display Server (GSDS2.0, http://gsds.cbi.pku.edu.cn, accessed on 27 June 2025) [73]. The *cis*-acting elements of *PutACBP* promoters were analyzed to further understand the *PutACBP* gene family. The 2500 bp upstream sequences of the *PutACBPs* promoter regions were downloaded FASTA format from the http://www.xhhuanglab.cn/data/alkaligrass.html (accessed on 27 June 2025) [48], and used to identify the putative *cis*-acting elements in PlantCARE (http://bioinformatics.psb.ugent.be/webtools/plantcare/html, accessed on 27 June 2025) [74]. The *cis*-acting elements were visualized on the GSDS 2.0 website. The conserved motifs in PutACBPs (with a maximum of 10 motifs) were predicted using the online MEME tools (http://meme-suite.org/tools/meme, accessed on 27 June 2025) [75].

### 4.6. Plant Materials, Growth Conditions, and Stress Treatments

The experimental materials for organ expression analysis were provided by Anda Experimental Base, Research Center for Biological Resources and Environment in Saline-Alkali Land, Northeast Forestry University. Roots, stems, leaves, sheaths, flowers, spikes, and seeds of wild alkaligrass from salt-alkali land were harvested, immediately frozen in liquid nitrogen, and then stored at −80 °C for further analysis.

For the specific expression experiment under various stresses, the cleaned seeds were soaked in darkness for 12 h, then evenly sown on the gauze of hydroponic culture and cultured in the greenhouse under long-day conditions (16 h light/8 h dark) at 25 °C, and supplied with water every two days. Seedlings that germinated after three weeks were subjected to the following conditions, 4 °C (cold), 200 mM NaCl, 100 mM NaHCO_3_, 75 mM Na_2_CO_3_, 10 mM H_2_O_2_, and 160 uM CdCl_2_, respectively. The control (untreated) and treated roots and leaves were harvested, respectively, at 0 h, 6 h, 12 h, 24 h and 48 h after treatments, respectively. All samples were frozen in liquid nitrogen and stored at −80 °C until further use.

The *35S::PutACBP3-GFP* was amplified with appropriate primers (Table S5) and cloned into the *pBI121* vector and then introduced into WT Via *Agrobacterium tumefaciens* (GV3101 strain) using the floral dip method [76]. Seeds were surface sterilized by fumigation with chlorine gas for 4 h, then plated on solidified half strength Murashige and Skoog (MS) medium. All the seeds were held for two days at 4 °C, and finally grown in a greenhouse delivering a 16 h photoperiod with a constant temperature of 22 °C.

### 4.7. RNA Extraction and Reverse Transcription Quantitative Polymerase Chain Reaction (RT-qPCR)

Total RNA was extracted from roots and leaves using Trizol reagent (9109, TaKaRa, Tokyo, Japan) following the manufacturer’s protocol. The quality of the RNA was determined using the NanoDrop 2000 spectrophotometer (Thermo Scientific, Waltham, MA, USA). After removing genomic DNA contamination with DNaseI, cDNA was synthesized by using the PrimeScriptTMRT Reagent Kit (RR092A, Takara, Tokyo, Japan). For RT-qPCR analysis, the ChamQ SYBR Color qPCR Master Mix (Q411, Vazyme, Nanjing, China) was used with *Putactin* and *PutGADPH* serving as an internal control. Primers of *PutACBP* genes were designed using Primer3 online tools (https://bioinfo.ut.ee/primer3-0.4.0/, accessed on 7 July 2025). All the primers were listed in Table S5. RT-qPCR analyses were performed using three independent biological replicates, and the data were normalized using the 2^−ΔΔCt^ method. The heat map of gene expression pattern was visualized using TBtools software.

### 4.8. Subcellular Localization of PutACBP and Microscopy Analysis

For subcellular localization analysis, the full-length CDS of *PutACBPs* were cloned into the vector *pBI121-GFP*, transformed into *A. tumefaciens* strain GV3101. The *A. tumefaciens* strain GV3101 containing *35S::PutACBPs-GFP* and *35S::GFP* constructions were incubated shaking until the OD_600_ reached 0.6–0.8, respectively. Centrifuge the cells at 4000× *g* for 5 min, then discard the supernatant and resuspend the pellet with induction buffer (0.01 M MES, 0.01 M MgCl_2_, 150 μM acetosyringone) to find OD_600_ = 0.6, inject the solution into three-week-old tobacco leaves. After 48 h incubation, the fluorescence signal of GFP was observed using a fluorescence microscope LSM 780 (Zeiss, Baden-Württemberg, Germany), with excitation at 488 nm.

### 4.9. Phenotype Analysis

The seedlings were grown on 1/2 MS for seven days then transferred to media supplemented with various concentration of NaCl. Position of root tips marked on the plate and grown for 10 days. The root lengths were determined from digital images using Image J software (v1.41). Fresh weight was also measured at the same time.

### 4.10. Nitroblue Tetrazolium (NBT) Staining

NBT staining was used to detect O^2·−^ using a method modified from that described by Dunand et al. [77]. Fifteen-day-old seedlings were treated with salt stress for two days. The untreated seedlings were used as experimental controls under the same conditions, then the seeding incubated in 2 mM NBT (Sigma, Darmstadt, Germany) in 20 mM K phosphate/0.1 M NaCl at pH 6.1 for 15 min, then transferred to distilled water.

### 4.11. Isolation of His-PutACBP3 Proteins from E. coli

The CDS of *PutACBP3* was amplified by PCR and cloned into *pET32a* expression vector. The construction was transformed into *Escherichia coli* strain BL21 (DE3) for protein expression. His-tagged proteins were purified using Ni-NTA resin according to instructions of the manufacturer.

### 4.12. Purification of Recombinant His-PutACBP3 for Filter-Binding Assays

The binding of His-PutACBP3 to the various lipids on the filters was performed as previously described [78] with minor modifications. Briefly, various concentrations of lipids were spotted onto nitrocellulose and incubated at room temperature for 1 h in dark. LysoPC, PC, PA, 18:0-PC, and 18:2-PC were purchased from Sigma, and 16:0-PC, 18:1-PC, and DMPC were purchased from Echelon Biosciences. The lipid-bound filter was blocked with Tris-buffered saline (TBS) with 1% nonfat milk for 1 h. After incubation with 1 μg·mL^−1^ purified His-ACBP3 protein in blocking buffer for 2 h, the filter was gently washed three times with TTBS (TBS plus 0.1% Tween 20), each for 10 min. Following incubation with the horseradish peroxidase (HRP)-conjugated anti-His antibodies (1:2000; Qiagen, Düsseldorf, Germany, Catalog No. 1014922 ) for 1 h at room temperature, the filter was again washed three times with TTBS, each for 10 min, and then detected with the ECL Western blotting Detection Kit (Amersham, Uppsala, Sweden) following the manufacturer’s protocols.

## 5. Conclusions

We identified five alkaligrass *ACBP* genes, which could be classified into four subfamilies. We confirmed that *PutACBP* genes displayed a great evolutionary divergence in alkaligrass by gene structure, conserved domains and phylogenetic analysis. Furthermore, we identified that *ACBP* genes showed special organ expression profiles, indicating their potential divergent functions in alkaligrass growth and development. *Cis*-acting elements analysis and expression profiles under different stresses suggested that ACBP family genes might be related to the responses of abiotic stresses and hormone stimuli. Overexpressing *PutACBP* gene in *Arabidopsis* seedlings revealed that *PutACBP3* was predominantly expressed in roots and mediated ROS homeostasis primarily under salinity stress. Moreover, we focused on the expression of *PLDs* in *Arabidopsis* overexpressing *PutACBP3*, the RT-qPCR showed that after 100 mM NaCl stress, a higher expression level in *PLDδ* than WT, suggesting that salt stress tolerance of *PutACBP3* may be related to the enhancement of *PLDδ* expression. Taken together, we established a foundation for further functional characterization of *PutACBP* genes in response to salinity stress in the future.

## Data Availability

Data is contained within the article and Supplementary Materials.

## Supplementary Materials

The following supporting information can be downloaded at https://www.mdpi.com/article/10.3390/plants14233551/s1, Figure S1: Predicted amino acid sequence for each motif. Motifs were predicted by MEME motif searching, and 10 were selected as the maximum number parameter; Figure S2: Induction and Purification of (His)_6_-PutACBP3 recombinant protein; Table S1: Features of *PutACBP* genes in *Puccinellia tenuiflora*; Table S2: List of coding sequences of *PuACBPs* from *Puccinellia tenuiflora*; Table S3: List of 86 ACBPs from *Puccinellia tenuiflora*, *Oryza sativa*, *Arabidopsis thaliana* and other Plants; Table S4: List of structural domain protein sequence from *Puccinellia tenuiflora*, *Oryza sativa*, and *Arabidopsis thaliana*; Table S5: List of primers used in cloning and RT-qPCR analysis for *PutACBPs*; Table S6: Organ- specific expression analysis of *PutACBPs*; Table S7: Expression analysis of *PutACBPs* in roots and leaves under differernt stress; Table S8: Expression profiles of *PLD* genes in *PutACBP3*-overexpressing *Arabidopsis* seedlings.
