# Supplementary material for: Genome-Wide Characterization, Expression, and Functional Analysis of Acyl-CoA-Binding Protein (ACBP) Gene Family in Puccinellia tenuiflora
Source: Plants (Basel). 2025 Nov 21;14(23):3551. doi: 10.3390/plants14233551 (PMC12694500; doi:10.3390/plants14233551)
Supplement: Supplementary file 1 [file plants-14-03551-s001.zip › Supplementary Files/Figure S1-S2.pdf]

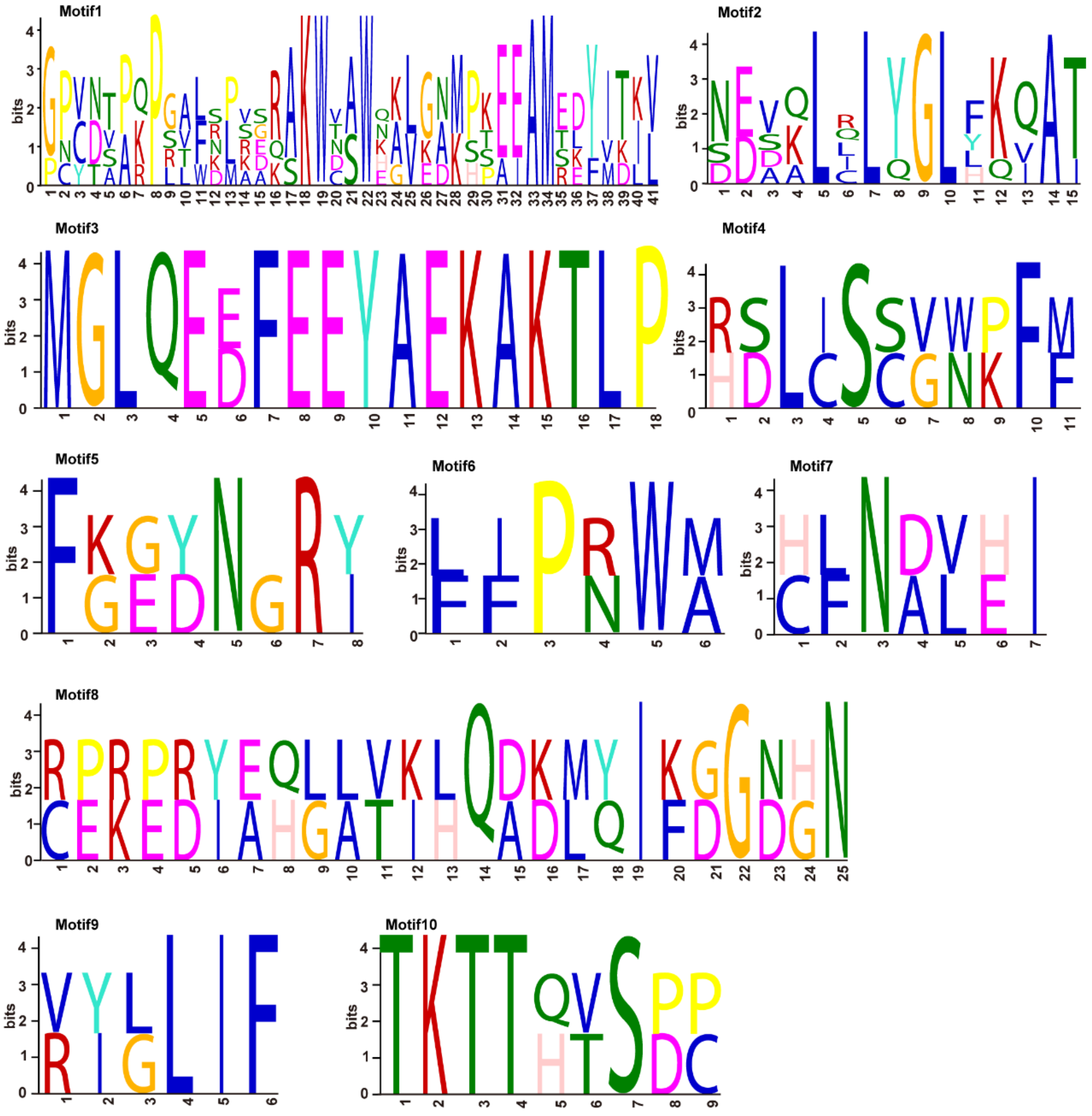

Figure S1 Predicted amino acid sequence for each motif. Motifs were predicted by MEME motif searching, and 10 were selected as the maximum number parameter.

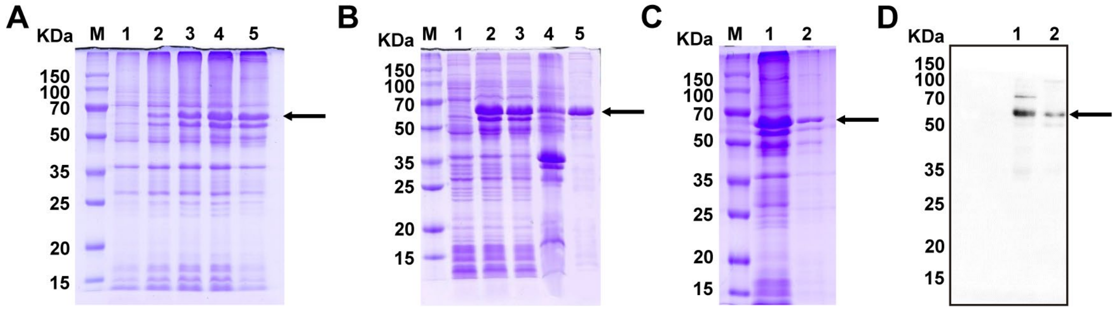

Figure S2 Induction and Purification of (His)$_6$-PutACBP3 recombinant protein.

(A) 10% sodium dodecyl sulfate–polyacrylamide gel electrophoresis (SDS–PAGE) gel shows the 54-kDa (His)$_6$-PutACBP3 protein induced from *E. coli* induced by isopropyl b-D-thiogalactoside (IPTG) for 0, 0.5, 1, 2, 4 h, respectively.

(B) Massive induction of (His)$_6$-PutACBP3. Lanes 1-5 are the pre-induction cells, the induced cells, the supernatant of the induced protein, the precipitation of the induced protein, and the purified protein, respectively.

(C) Purification of (His)$_6$-PutACBP3 recombinant protein in *E. coli.* Lanes 1-2 are the supernatant of the induced protein, the purified (His)$_6$-PutACBP3 protein.

(D) Western blot analysis of (His)$_6$-PutACBP3, Lanes 1-2 are immune blot analysis of the (His)$_6$-PutACBP3 protein.

M: Protein marker; The arrow indicates the target protein.
